# Bovine ephemeral fever virus triggers autophagy enhancing virus replication via upregulation of the Src/JNK/AP1 and PI3K/Akt/NF-κB pathways and suppression of the PI3K/Akt/mTOR pathway

**DOI:** 10.1186/s13567-019-0697-0

**Published:** 2019-10-10

**Authors:** Ching-Yuan Cheng, Hsu-Hung Tseng, Hung-Chuan Chiu, Ching-Dong Chang, Brent L. Nielsen, Hung-Jen Liu

**Affiliations:** 1 Institute of Molecular Biology, National Chung Hsing University, Taichung, 402 Taiwan; 2 Division of General Surgery, Taichung Hospital, Ministry of Health and Welfare, Taichung, 402 Taiwan; 3 The iEGG and Animal Biotechnology Center, National Chung Hsing University, Taichung, 402 Taiwan; 4 Department of Veterinary Medicine, National Pingtung University of Science and Technology, Pingtung, Taiwan; 5 Department of Microbiology and Molecular Biology, Brigham Young University, Provo, UT USA; 6 Rong Hsing Research Center for Translational Medicine, National Chung Hsing University, Taichung, 402 Taiwan; 7 Ph.D. Program in Translational Medicine, National Chung Hsing University, Taichung, 402 Taiwan; 8 Department of Life Sciences, National Chung Hsing University, Taichung, Taiwan

## Abstract

Autophagy plays an important role in cellular response to pathogens. However, the impact of the autophagy machinery on bovine ephemeral fever virus (BEFV) infection is not yet determined. A recent study in our laboratory demonstrated that BEFV triggers simultaneously the PI3K/Akt/NF-κB and Src/JNK-AP1 pathways in the stage of virus binding to enhance virus entry. In this work, we report that BEFV induces autophagy via upregulation of the PI3K/Akt/NF-κB and Src/JNK/AP1 pathways in the early to middle stages of infection and suppresses the PI3K/Akt/mTOR pathway at the late stage of infection. To activate NF-κB, BEFV promotes degradation of IκBα and activates Akt to stimulate NF-κB translocation into the nucleus. Immunoprecipitation assays revealed that BEFV disrupts Beclin 1 and Bcl-2 interaction by JNK-mediated Bcl-2 phosphorylation, thereby activating autophagy. Overexpression of Bcl-2 reversed the BEFV-induced increase in the LC3 II levels. Suppression of autophagy either by knockdown of autophagy-related genes with shRNAs or treatment with a pharmacological inhibitor 3-MA reduced BEFV replication, suggesting that BEFV-induced autophagy benefits virus replication. Our results revealed that the BEFV M protein is one of the viral proteins involved in inducing autophagy via suppression of the PI3K/Akt/mTORC1 pathway. Furthermore, degradation of p62 was observed by immunoblotting, suggesting that BEFV infection triggers a complete autophagic response. Disruption of autophagosome-lysosome fusion by depleting LAMP2 resulted in reduction of virus yield, suggesting that formation of autolysosome benefits virus production.

## Introduction

Being a multifunctional protein, the mammalian target of rapamycin (mTOR) interacts with different partner proteins to regulate distinct signaling cascades. The mTOR complex 1 (mTORC1) is composed of mTOR, raptor, and GβL. mTORC1 activity is controlled by multiple signaling pathways including the PI3K/Akt and adenosine 5′-monophosphate (AMP)-activated protein kinase (AMPK) pathways [1, 3]. Phosphatidylinositol 3-kinases (PI3Ks) are a family of lipid kinases. Activated PI3K phosphorylates phosphoinositides at the 3′-position of the inositol ring to generate the major lipid product, phosphatidylinositol 3,4,5-triphosphate (PIP_3_), which recruits downstream factors to the cell membrane domains [3]. Akt, an essential downstream factor of PI3K, is up-regulated by phosphoinositide-dependent kinase 1 (PDK1)-mediated phosphorylation at T308 after recruitment [2–4]. Concurrent phosphorylation at Thr308 by PDK1 and at S473 by mTORC2 is required for full activation of Akt [5, 6]. More recently, it was demonstrated that phosphorylation of Akt at S477 and T479 by the cyclin-dependent kinase 2 (CDK2)/cyclin A complex enhances Akt activation by functionally compensating for Akt S473 phosphorylation [7, 8]. Activation of the PI3K/Akt pathway up-regulates the activity of mTORC1. Unlike PI3K-Akt signaling, AMPK is a negative regulator of mTORC1. Besides functioning as a sensor of cellular energetic stress, AMPK activates tuberous sclerosis complex 2 (TSC2), a GTPase-activating protein that forms a complex with TSC1, and stimulates the intrinsic GTPase activity of Rheb which eventually results in mTORC1 inactivation [9]. Activation of mTORC1 facilitates the function of a few translation initiation factors through governing downstream factors like eIF-4E binding protein 1 (4E-BP1). In the absence of external stimuli, 4E-BP1 sequesters eIF-4E preventing initiation of cap-dependent translation. Phosphorylated 4E-BP1 dissociates from eIF-4E, allowing for the binding of eIF4E to eIF4G, thereby facilitating the assembly of the initiation complex eIF4F and subsequent translation [3].

The PI3K/Akt pathway is an important signaling pathway through which viral infection affects various cell functions. Many pathogens are known to up-regulate the PI3K/Akt pathway for their efficient replication or persistence in the host [10]. A few persistently-infecting viruses activate the PI3K/Akt/mTOR pathway to maintain long-term infection [11]. Many viruses have been demonstrated to activate the PI3K/Akt pathway enhancing virus entry [12–16]. It has recently been shown that Akt activity is important for RNA synthesis of non-segmented, negative-stranded RNA viruses [17].

Bovine ephemeral fever virus (BEFV), an arthropod borne virus, is a member of Rhabdoviridae. The bullet-shaped virion consists of a single stranded, negative-sense RNA genome with a lipid envelope and five structural proteins, including the large RNA-dependent RNA polymerase (L), the polymerase-associated protein (P), the envelope glycoprotein (G), the nucleoprotein (N), and the matrix proteins (M) [18, 19]. As is the case for several enveloped RNA viruses, the M protein of rhabdoviruses is critical for virus assembly and budding. In the absence of other viral products, M protein alone is able to bud from cell surfaces in the form of lipid-enveloped, virus-like particles (VLPs) [20, 21]. Our team has demonstrated that BEFV triggers simultaneously the PI3K/Akt/NF-κB and Src/JNKAP1 pathways at virus binding stage to enhance virus entry [12]. Our earlier study demonstrated that BEFV activates Akt and inhibits mTORC1 to benefit BEFV replication [10]. Nevertheless, the underlying mechanisms of how BEFV ingeniously inhibits mTORC1 but up-regulates Akt to support its multiplication remains poorly understood. In this study, we further demonstrate that BEFV induces autophagy via upregulation of the PI3K/Akt/NF-κB and the Src/JNK/AP1 pathways in the early to middle stages of infection and causes suppression of the PI3K/Akt/mTORC1 pathway at the late stage of infection, all of which benefit virus replication. We also found that the BEFV M protein plays an important role in autophagy induction via suppression of the PI3K/Akt/mTOR pathway.

## Materials and methods

### Cells and viruses

Madin-Darby bovine kidney (MDBK) cells and baby hamster kidney (BHK 21) cells were purchased from Sigma-Aldrich Co. (St. Louis, USA). The BHK-21 cells stably expressing human Bcl-2 (BHK 21/Bcl 2) were described previously [22]. Cells were cultured in Dulbecco’s modified eagle medium (DMEM) supplemented with 10% fetal bovine serum (FBS). One day before each experiment, cells were seeded in 6-cm cell culture dishes with 1 × 10^6^ cells, and incubated at 37 °C with 5% CO_2_. The 2004/TW/TN1 strain of BEFV was propagated in BHK-21 cells. When 70–80% cytopathic effect (CPE) was shown, the supernatants of BEFV-infected cells were harvested, condensed by PEG 6000 precipitation, dialyzed and resuspended in phosphate-buffered saline (PBS), and finally stored at −70 °C before use.

### Antibodies

The primary antibodies used in this study are shown in Table 1. The catalog numbers and dilution factor of the antibodies used in this study are shown in Table 1. Polyclonal antibodies against the BEFV M protein are from our laboratory stock. Anti-rabbit IgG (H + L) and anti-mouse IgG (H + L) antibodies were purchased from Kirkegaard & Perry Laboratories (Washington, DC., USA).

**Table 1:** **The catalog numbers and dilution factor of the respective antibodies used in this study**

Antibodies	Catalog number	Clone name	Dilution factor	Manufacture
Mouse anti-M	–	–	2000	Our laboratory
Rabbit anti-p-mTOR (S2448)	2971	ND	1500	Cell Signaling
Rabbit anti-mTOR	2983	7C10	3000	Cell Signaling
Rabbit anti-p-PI3Kp85 (Y458)	4228	ND	2000	Cell Signaling
Rabbit anti-PI3K p85	4257	19H8	2000	Cell Signaling
Rabbit anti-p-4E-BP1 (T37/46)	2855	236B4	1500	Cell Signaling
Rabbit anti-4E-BP1	9644	53H11	2000	Cell Signaling
Rabbit anti-p-Akt (S308)	2965	C31E5E	3000	Cell Signaling
Rabbit anti-p-Akt (S473)	3787	736E11	2000	Cell Signaling
Rabbit anti-Akt	2964	5B5	3000	Cell Signaling
Rabbit anti-p-ULK1 (S757)	6888	ND	1500	Cell Signaling
Rabbit anti-ULK1	4773	R600	3000	Cell Signaling
Rabbit anti-Atg7	8558	D12B11	3000	Cell Signaling
Rabbit anti-Beclin 1	3495	D40C5	1500	Cell Signaling
Mouse anti-IkBα	4814	L35A5	3000	Cell Signaling
Rabbit anti-p65	4764	C22B4	2000	Cell Signaling
Rabbit anti-p50	3035	ND	2000	Cell Signaling
Rabbit anti-histone H2A	2578	ND	2000	Cell Signaling
Rabbit anti-LAMP2	49 067	D5C2P	2000	Cell Signaling
Mouse anti-p-Bcl-2	05-613	ND	1500	Upstate
Mouse anti-Bcl-2	15 071	124	3000	Cell Signaling
Rabbit anti-p62	7695	D10E10	2000	Cell Signaling
Rabbit anti-p-Src (Y416)	2113	100F9	1500	Cell Signaling
Mouse anti-Src	2110	L4A1	3000	Cell Signaling
Rabbit anti-p-SAPK/JNK (T183/Y185)	9251	ND	2000	Cell Signaling
Rabbit anti-SAPK/JNK	9252	ND	3000	Cell Signaling
Mouse anti-β-actin	sc-47778	C4	5000	Santa Cruz
Rabbit anti-LC3B	2775	ND	3000	Cell Signaling

### Chemical inhibitors and reagents

To investigate the signaling pathways mediating BEFV-induced autophagy, the chemical inhibitors and reagents specific for signaling molecules of interest were used in this study. Rapamycin, 3-methyladenine (3-MA), Bay11-7085, and Tanshinone were purchased from Calbiochem Co. (San Diego, USA). Thapsigarigin (TG) was purchased from Sigma-Aldrich Co. Akt III was purchased from Enzo Life Science (New York, USA).

### Specific gene-silencing shRNA constructs

All shRNAs and scrambled negative shRNA (TR30013) were constructed in the pGFP-V-RS (TR30007) plasmid from OriGene Co. (Rockville, USA). After preliminary tests, the shRNA constructs exhibiting the most significant suppression effect on the expression of the target gene were used in this study. The shRNAs are shown in Table 2. Transfection of cells with plasmid DNA was carried out using TurboFect™ in vitro transfection reagent (Thermo Fisher Scientific, Waltham, USA) according to the manufacturer’s suggestions. The transfected cells were then infected with BEFV of at an multiplicity of infection (MOI) of 1 at 24 h post-transfection for further research purposes.

**Table 2:** **shRNAs used in this study**

Target gene	Cat. no	Tube ID	Target sequence (5′–3′)
Akt	TRCN0000010163	–	CGAGTTTGAGTACCTGAAGCT
Atg7	TG314609	GI358429	TGCCAGCTCGCTTAACATTGGAGTTCAGT
LC3-II	TG503902	GI515131	TGGACAAGACCAAGTTCCTGGTGCCTGAC
LAMP2	TG311794	GI347172	CCAATTATAGTTGGTGCTGGTCTTTCAGG
Beclin 1	TG314484	GI357930	TGTCAGAACTACAAACGCTGTTTGGAGAT

### Cell viability assay

To examine whether the shRNAs and compounds used in this study have deleterious effects on the cell, cell viability was examined by MTT assay. MDBK cells were seeded in 4-well plates 1 day before this procedure. At about 60% confluence, cells were transfected with the respective shRNA or treated with compounds, respectively. Twenty-four hours later, 50 μL of thiazolyl blue tetrazolium bromide (MTT; Sigma-Aldrich) were added to each well. Cells were swirled gently for a few seconds and cultured for a further 3 h. The medium was removed and the cells were washed twice with PBS. After gentle swirling for a few minutes, the optical density of 50 μL of supernatant was evaluated at 570 nm, with subtraction of background at 670 nm.

**Figure 1:**
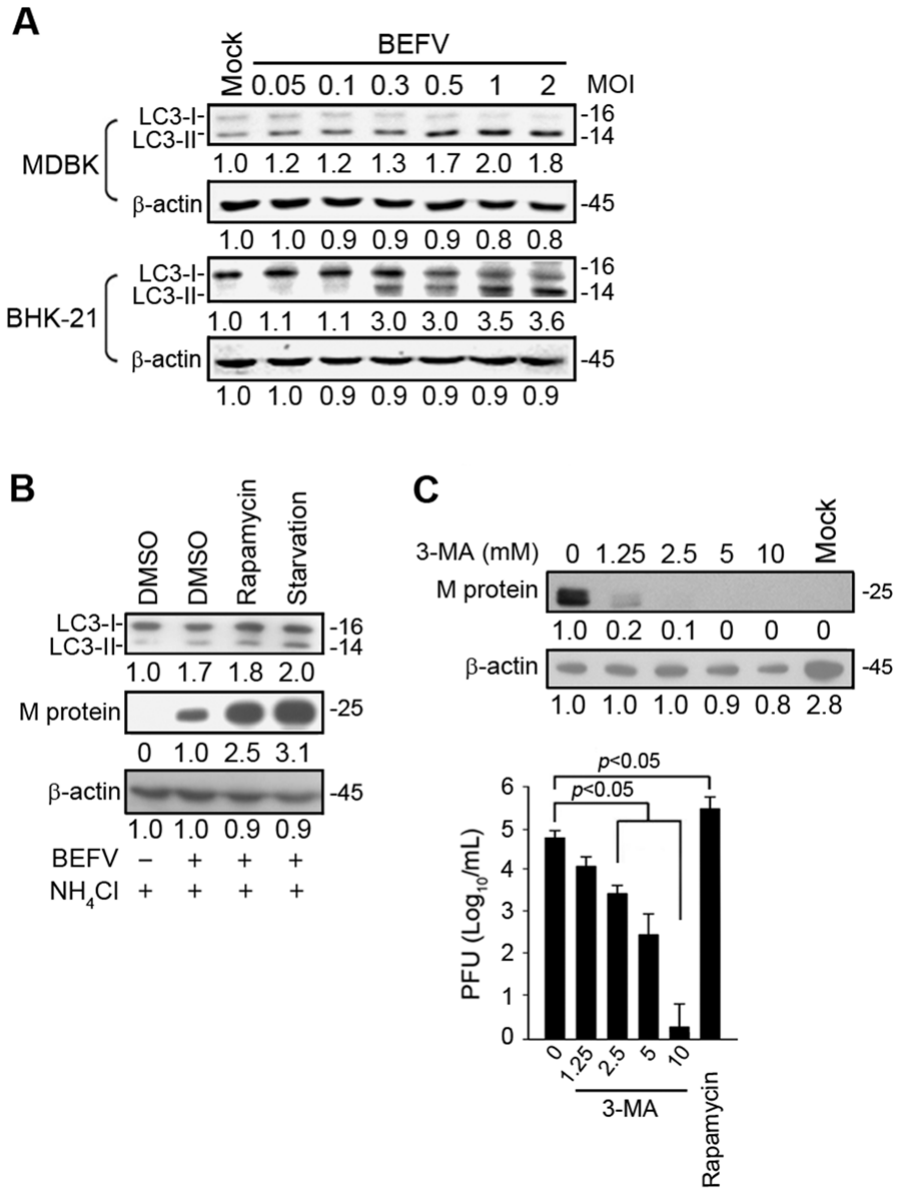
**BEFV induces autophagy benefiting virus replication. A** MDBK and BHK-21 cells were infected with BEFV at different MOIs for 18 h and the levels of LC3-II were determined. **B** MDBK cells were seeded in 6-well cell culture dishes with 2 × 10^5^ cells. BEFV-infected MDBK cells at a MOI of 1 for 18 h were then treated with NH_4_Cl (20 mM) alone or NH_4_Cl (20 mM) combined with rapamycin (5 µM) or starvation (cells supplemented with no FBS), and the cell lysates were collected individually at 18 h post-infection (hpi) for immunoblotting with the respective antibodies. **C** Cells were pretreated with rapamycin (5 µM) and 3-MA (1.25, 2.5, 5, and 10 mM), respectively, for 30 min and then infected with BEFV at an MOI of 1 for 18 h. The effects of rapamycin and 3-MA on BEFV production were determined by analyzing the expression level of the BEFV M protein (upper panel) and virus titer (lower panel). All data shown represent the mean ± SD calculated from three independent experiments. Signals in all Western blots were quantified with Image J software. The levels of indicated proteins in the mock control (mock, DMSO or un-treatment) were considered onefold. The protein levels were normalised to those for β-actin. The activation and inactivation folds indicated below each lane were normalized against values for the mock control. The predicated size of each protein was labeled at the right-hand side in kDa.

### Virus titration

The supernatant of BEFV-infected cells at 24 h post-infection (hpi) was collected to determine the virus titer. The supernatant containing BEFV particles was serially diluted with serum-free DMEM. Each serial diluted virus solution (200 μL) was incubated with MDBK cells seeded in a 24-well-plate for 1 h. The cells were washed with phosphate buffered saline (PBS) buffer to remove unabsorbed viruses and overlaid with 0.6 mL of 0.8% agarose in DMEM containing 2% FBS. Plaques formed by BEFV were counted after an incubation period of 2 to 3 days at 37 °C by staining with neutral red for 3 h.

### Isolation of the protein fractions of cytoplasm and nucleus

Cellular protein fractions from the different samples were extracted using the CNM compartmental Protein Extraction Kit (Biochain Institute Inc., Hayward, USA) following the instructions of the manufacturer. Briefly, cells were suspended in ice-cold buffer C provided in the kit, cell homogenization was then carried out by passing cells through a 1-mL syringe with a bended 26.5 gauge needle. After centrifugation at 15 000 *g* at 4 °C for 20 min, the supernatant containing cytoplasmic proteins was removed and placed in another tube. The pellet was washed with cold buffer W, which was removed after centrifugation at 15 000 *g* at 4 °C for 20 min, followed by resuspending the pellet with cold buffer N to extract the nuclear proteins. After centrifugation at 15 000 × *g* at 4 °C for 20 min, the supernatant containing nuclear proteins was transferred to a clean tube.

### Electrophoresis and Western blot

Cells were washed twice with PBS and lysed in 70 μL 2.5× Laemmli loading buffer (200 mM Tris, pH 6.8; 8% SDS, 10% β-mercaptoethanol, 40% glycerol, 0.04% bromophenol blue). After scraping, the cell lysates were collected and boiled for 10 min. Equal amounts of samples were run on 10% or 15% sodium dodecyl sulphate–polyacrylamide gel electrophoresis (SDS-PAGE) and then transferred to PVDF membranes. Expression of individual proteins was detected using their respective antibodies, followed by a secondary antibody conjugated with horseradish peroxidase (HRP). After appropriate washes of the membranes with TBST buffer (50 mM Tris–HCl pH7.5, 150 mM NaCl and 0.1% Tween 20), the membranes were soaked with enhanced chemiluminescence solution (ECL plus) (Amersham Biosciences, Little Chalfont, Buckinghamshire, UK) and then exposed to X-ray film. The intensity of each protein was calculated using the program Photocapt (Vilber Lourmat, France). Signals in all Western blots were quantified with Image J software [23]. The levels of indicated proteins in the mock control were considered onefold. The protein levels were normalised to those for β-actin. The activation and inactivation folds indicated below each lane were normalized against values for the mock control.

### Plasmid construction

mCherry is relatively stable. In order to dissect cellular LC3 puncta during the process of BEFV-induced autophagy, a DNA segment containing gfp-mCherry-LC3 coding sequences was cloned into *Nhe*I and *Sma*I sites of the pCI-neo mammalian expression vector (Promega, Madison, USA). The resultant plasmid was transfected into MDBK cells to express GFP-mCherry-LC3 fusion protein. Due to the pH instability of GFP, it will lose its fluorescence when the autophagosome fuses with the lysosome. Thus, GFP-mCherry-LC3 could be an alternative autophagy marker to detect autophagosomes and autolysosomes. To prepare cDNA for the BEFV M gene, total RNA was extracted from BEFV-infected MDBK cells using TRIzol solution (Thermo Fisher Scientific Inc.), followed by conducting reverse transcription reaction using M-MLV RT (Promega, Madison, USA) according to the manufacturer’s protocol. Reverse transcription was carried out at 42 °C for 90 min. 2 µg of total RNA was used as template and mixed with 0.5 mg oligo (dT) in a total volume of 14 µL in water. After heating at 70 °C for 5 min, the tube was quickly placed on ice and 11 µL of reaction solution containing 5 µL of M-MLV 5 × reaction buffer, 5 µL of 2.5 mM dNTPs, and 1 µL of M-MLV reverse transcriptase (200 units) was added. The reverse transcription product was used as template to amplify the M gene of BEFV by polymerase chain reaction (PCR) with 0.5 µL of *pfu* DNA Polymerase (MD Bio Inc., Taiwan), 2 µL of 2.5 mM dNTP, 5 µL of 10× *Pfu* buffer and 1 µL of 10 µM primers (Forward: GCTAGCATGCTTACCCTTTTCAAGAAAGG and reverse: CTCGAGTCATGAC TTAACTAAGTTAGTGA) and adjusted with nuclease-free water to a final volume of 50 µL. The PCR condition is as follows: one cycle of 94 °C for 3 min; 35 cycles of 94 °C for 40 s, 45 °C for 1 min, 72 °C for 1 min; one cycle of 72 °C for 15 min. The amplified PCR products is 672 bp in length. After cleavage by restriction enzymes, the PCR product containing the M gene coding sequences was cloned into the NheI and SalI sites of the pCI-neo-based vector to generate pCI-Flag-M, which expresses Flag-tagged BEFV M protein.

### Fluorescence microscope and image acquisition and analysis

MDBK cells expressing GFP-mCherry-LC3 proteins were seeded on slide coverslips (18 × 18 mm) for 24 h for fully attachment. To dissect the effects of the chemical agents on BEFV-induced autophagy, the medium was replaced by a new one containing 2% FBS and the chemical agent was added prior to BEFV infection. After 30 min cells were infected by BEFV at a MOI of 1 for the indicated times and then fixed with 4% paraformaldehyde in PBS at room temperature for 1 h. Cells were soaked in PBS with 0.3% Triton X-100 at room temperature for 10 min, washed with PBS, and then blocked with SuperBlock^®^ T20 (PBS-based) blocking buffer (ThermoFisher Scientific, USA) at 4 °C for 30 min. Cell nuclei were stained with 4′,6-diamidino-2-phenylindole (DAPI) for 10 min and washed three times with PBS at room temperature for 10 min each. The coverslips were mounted onto glass slides. The ibidi mounting medium (ibidi GmbH, Germany) was used to mount the coverslips onto glass slides. The cellular location of LC3 was observed using a fluorescence microscopy. To acquire images, Viewerfinder Lite sofeware (Pixera Corp., Santa Clara, USA) was used. Adobe photoshop CC was used to make image montage.

### Co-immunoprecipitation (Co-IP)

Cells were seeded and cultured in a 6-well plate until the cell confluence reached 70–80%. Virus infection was performed by adding BEFV (1 MOI) into the culture medium for 6 h. Cells were then washed with PBS and lysed with 1 × cell lysis buffer (Cell Signaling Technology; Danvers, USA). Immunoprecipitation of extracted total cellular protein (500 μg) with 9.1 µL of Beclin 1 (111 µg/mL) and 4.2 µL of Bcl-2 (237 µg/mL) antibodies, respectively, was performed using a Pierce Co-Immunoprecipatation Kit (ThermoFisher Scientific, USA) according to the protocol provided by the manufacturer. The eluted protein fractions were subjected to SDS-PAGE and the protein level of Beclin 1 and Bcl-2 were analyzed by Western blot.

### Statistical analysis

All data obtained in this study were analyzed using an independent sample *t* test and expressed as averages of three independent experiments. *P* values of less than 0.05 were considered significant.

## Results

### BEFV triggers autophagy and promotes virus growth

To explore whether BEFV induces autophagy, Madin-Darby bovine kidney (MDBK) and BHK 21 cells were infected with BEFV at different MOI for 18 h. To determine the levels of LC3 I/II, which is regarded as a hallmark protein for autophagy [24], cell lysates were collected for Western blot assays. The results presented in Figure 1A showed that levels of LC3-II increased after BEFV infection in both cell lines in a dose-dependent manner, indicating that BEFV induces autophagy.

To investigate the role of autophagy in BEFV replication, we examined the effects of rapamycin and starvation on autophagosome induction. Autophagy can be pharmacologically induced by inhibiting mTORC1 with rapamycin [25]. As shown in Figure 1B, rapamycin treatment or starvation increased the levels of LC3-II (Figure 1B). Treatment with rapamycin increased viral protein synthesis and virus yield (Figures 1B and C). Autophagy can be pharmacologically inhibited with a widely used selective inhibitor of autophagy, 3-MA, by targeting the class III PI3K, which involves autophagosome formation [26]. To determine whether autophagosome induction during BEFV infection was a host antiviral response or a viral replication mechanism, the effect of 3-MA on BEFV replication was further investigated. Treatment with 3-MA resulted in a dose-dependent reduction in the level of M protein and virus yield (Figure 1C).

To investigate whether compounds or shRNAs used in this study have deleterious effects on the cell, viability of the cells was examined and shown in Additional file 1. Cell viability in drug-treated cells was only slightly reduced as compared to mock or DMSO treatment. Taken together, our findings suggest a critical role of autophagy in the virus life cycle.

### Knockdown of the genes crucial for autophagosome formation reduced virus yield

**Figure 2:**
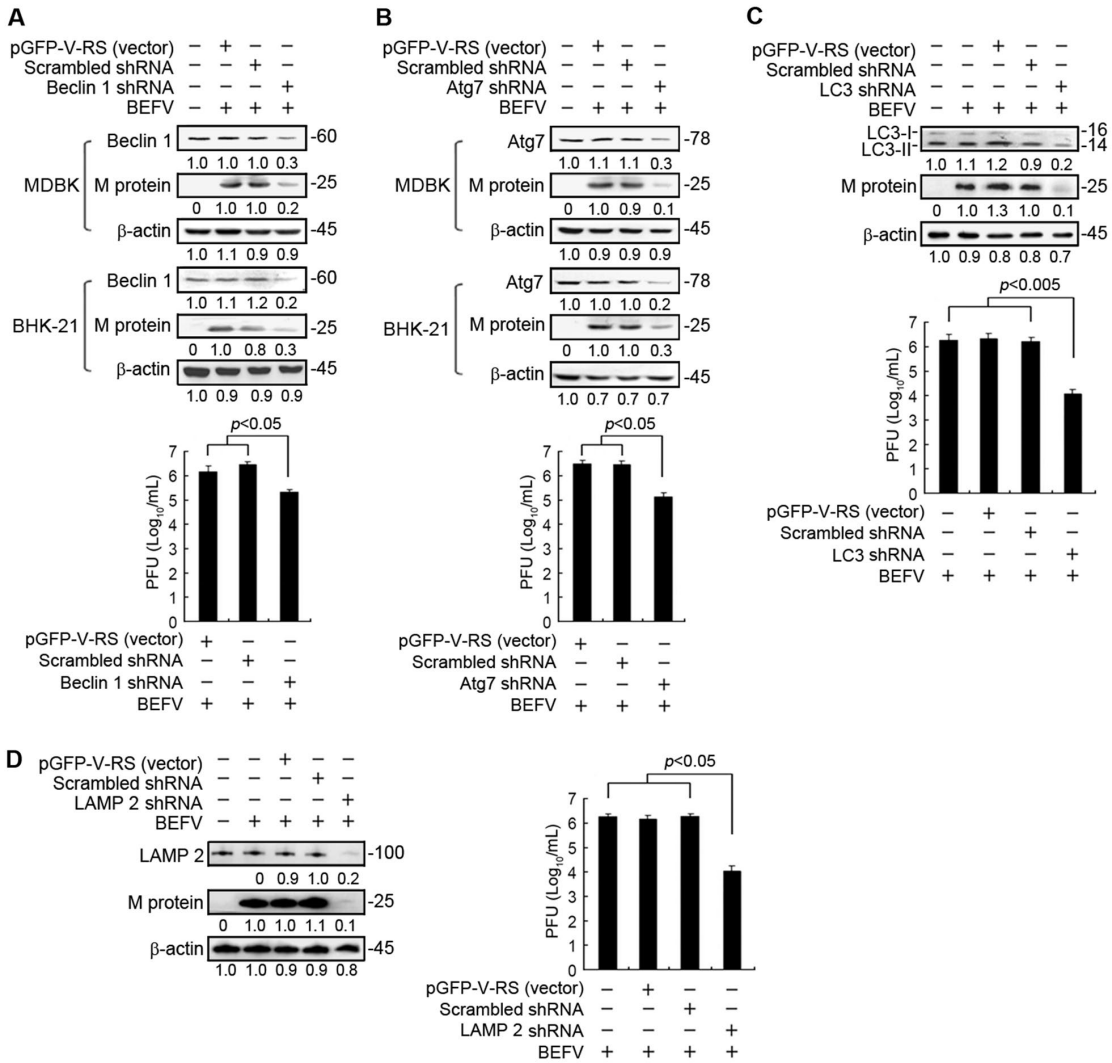
**Depletion of autophagy-related proteins reduced BEFV production.** MDBK or BHK-21 cells were transfected with autophagy-related gene shRNAs including **A** Beclin 1, **B** Atg7, **C** LC3, and **D** LAMP2, respectively for 24 h followed by infection with BEFV at a MOI of 1 and collected at 24 hpi. The expression level of the BEFV M protein in cell lysates was determined by Western blot and the culture medium was used for virus titration assays. All data shown represent the mean ± SD calculated from three independent experiments. Signals were quantified using Image J software. The levels of indicated proteins in the mock controls (un-infection or un-treatment) were considered onefold. The protein levels were normalised to those for β-actin. The activation and inactivation folds indicated below each lane were normalized against values for the mock control. The predicted size of each protein was labeled at the right-hand side in kDa.

Having demonstrated that autophagy is induced during BEFV infection, we further investigated the necessity of cellular autophagy for BEFV production. To this end, we attempted to suppress cellular expression of Atg-related genes via shRNA technology. Transfection of MDBK or BHK-21 cells with shRNAs that specifically target Beclin 1, Atg7, LC3, and LAMP 2 (Figures 2A–D), respectively, was carried out. To investigate whether shRNAs used in this study have deleterious effects on the cell, viability of the cells was examined and shown in Additional file 1B. Cell viability in shRNA-depleted cells was only slightly reduced as compared to mock or vector alone. Our results showed that the disruption of the class III PI3K signaling complex required for autophagosome formation by Beclin 1 shRNA resulted in significant reduction in M protein synthesis and viral yield (Figure 2A). As shown in Figure 2B, depletion of ATG7 blocked viral protein synthesis and viral yield (Figure 2B). The autophagy protein ATG7 is essential for activation of autophagosome formation and maturation machinery [27, 28]. Depletion of LC3 also significantly blocked viral protein synthesis and viral yield (Figure 2C). Furthermore, we examined whether prevention of autophagosome-lysosome fusion affects viral replication. LAMP2 is critical for autophagosome-lysosome fusion [29]. In this study, our results revealed that depletion of LAMP2 resulted in a decrease of viral protein synthesis and virus yield (Figure 2D). Because blocking the fusion between autophagosomes and lysosomes reduces viral yield, this suggests that autophagosome maturation likely plays a critical role in BEFV replication.

### BEFV delays cellular autophagy flux during infection

**Figure 3:**
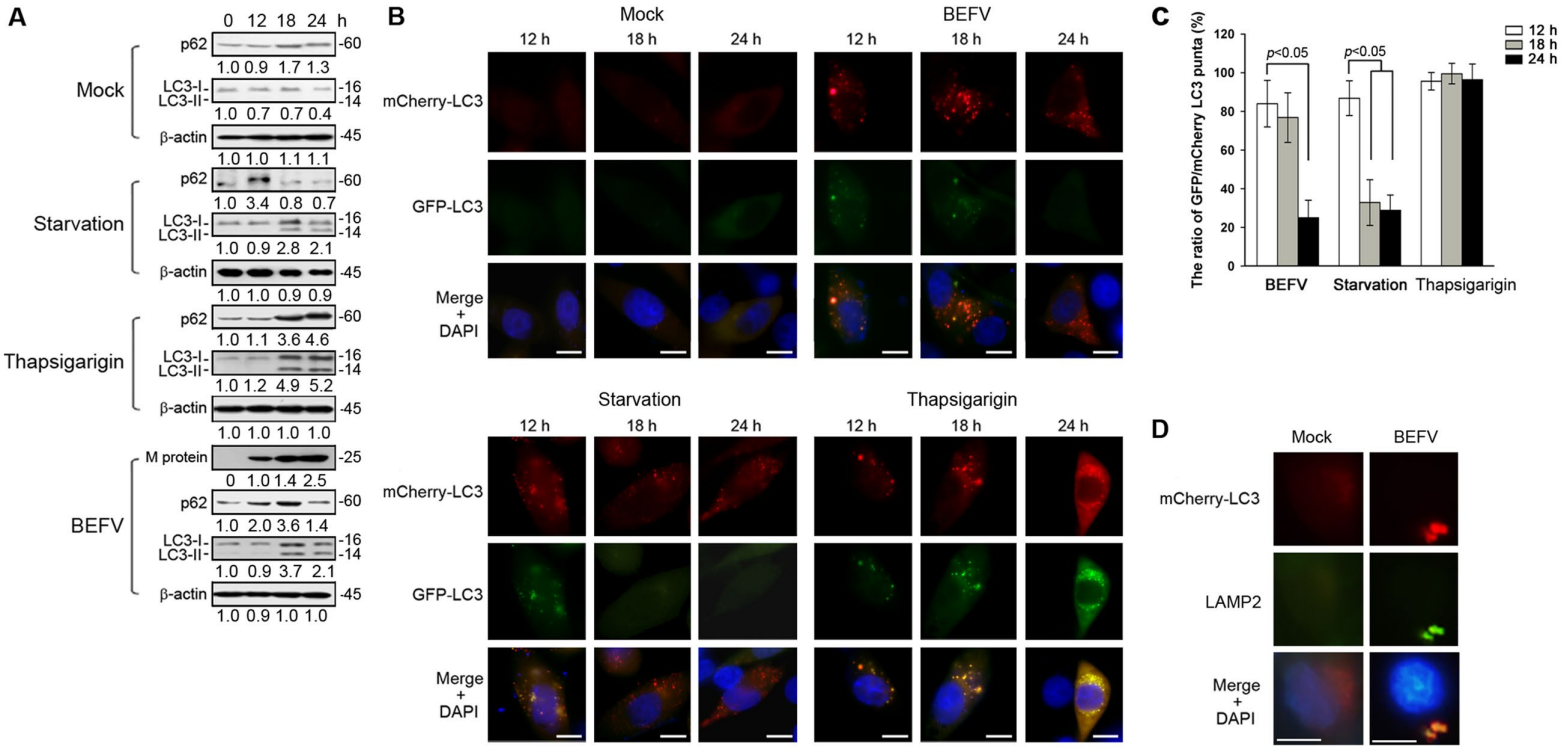
**BEFV promotes autophagosome formation. A** MDBK cells were respectively infected with BEFV at a MOI of 1, starved (cells supplemented with no FBS), or treated with thapsigarigin (10 µM). The cell lysates were collected at the indicated time points and subjected to immunoblotting with the respective antibodies. Signals were quantified using Image J software. The levels of the indicated proteins at 0 h were considered onefold. The protein levels were normalised to those for β-actin. The activation and inactivation folds indicated below each lane were normalized against values for the 0 h. The predicated size of each protein was labeled at the right-hand side in kDa. **B** LC3 puncta were observed under the fluorescence microscope. Cell nuclei were stained by DAPI. Scale bars, 25 µm. Normalization of the GFP/mCherry-LC3 puncta (%) were presented in **C**. **D** MDBK cells were transfected with mCherry-LC3 plasmid for 24 h and then infected with BEFV at MOI of 1 for 18 h. The cells were then fixed and processed for immunofluorescence staining of mCherry-LC3 and LAMP2. The colocalization of LAMP2 and LC3 was observed under a fluorescence microscope. Scale bar, 25 µm.

To further investigate autolysosome formation in BEFV infection, we next monitored cellular autophagy flux upon BEFV infection. BEFV-infected cell lysate was collected at the indicated time points and the levels of autophagic protein markers including p62 and LC3 were analyzed. p62 is a multifunctional protein that interacts with LC3-II, and is selectively degraded by the autophage-lysosomal pathway [30]. The p62 protein has also been considered as a marker for autophagy-mediated protein degradation or autophagic flux. A commonly used method to detect autophagic flux involves monitoring the levels of polyubiquitin-binding protein p62. The expression level of p62 increased at 18 hpi and then decreased at 24 hpi as compared with starvation or thapsigarigin treatments (Figure 3A). This suggests that BEFV may prevent p62 from degradation through an unknown mechanism before completion of virus replication. In contrast, during starvation-induced autophagy, autophagosomes fuse with lysosomes, leading to the degradation of intra-autophagosomal LC3-II and p62 by lysosomal proteases (Figure 3A). Furthermore, cells pretreated with TG inhibited formation of autolysosome and enhanced the accumulation of p62 and LC3 II (Figure 3A). Our result demonstrated that the half-life of p62 was extended in BEFV-infected cells, implying that lysosomal degradation was inhibited between 12 and 18 hpi. To confirm this observation, we utilized a pH-sensitive GFP-mCherry-LC3 reporter plasmid to dissect the maturation process of autophagosomes by performing immunofluorescence cell staining. GFP-LC3 is unstable in the lysosomal acidic and degradative conditions [31], while mCherry-LC3 is relative stable. Taking advantage of this property, the ratio of GFP/mCherry LC3 puncta can indicate whether or not the autophagic compartments fuse with lysosomes (Figure 3C). Under starvation condition, GFP-LC3 lost fluorescence at/after 18 h, whereas the GFP-LC3 signal remained in BEFV-infected cells until 18 hpi. Furthermore, the colocalization of mCherry-LC3 and LAMP2 was observed under a fluorescence microscope (Figure 3D). Taken together, our results suggest that BEFV induces autolysosome formation without enhancing autophagic protein degradation before 18 hpi.

### BEFV triggers autophagy via activation of the PI3K/Akt/NF-κB pathway

**Figure 4:**
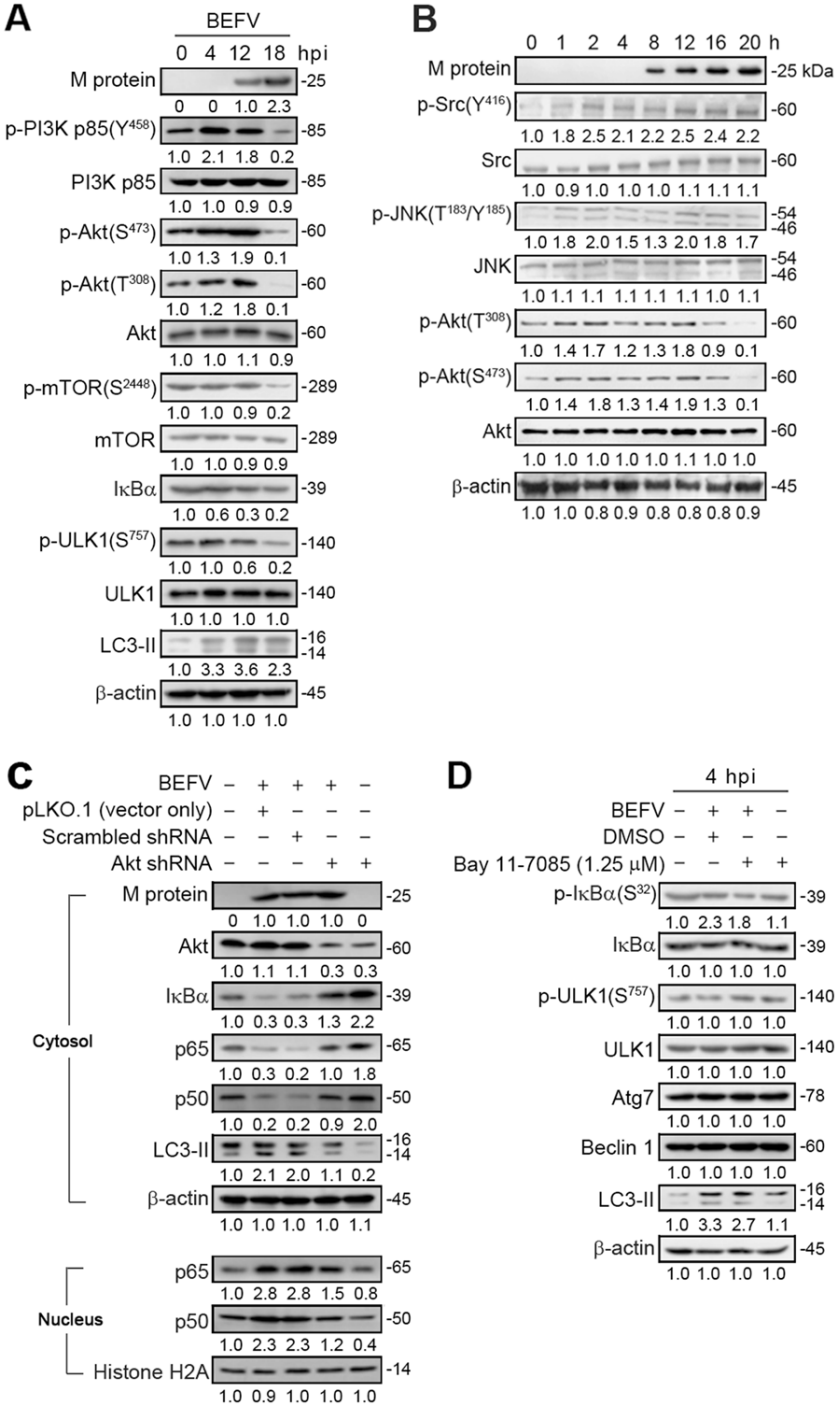
**BEFV induces autophagy through upregulation of the PI3K/Akt/NF-κB pathway in the early to middle stages. A**, **B** MDBK cells were infected with BEFV at a MOI of 1 and the cell lysates were collected individually at the indicated time points for immunoblotting with the indicated antibodies. **C** The levels of Akt, IkBα, p65, p50, and LC3-II were examined in BEFV-infected cells transfected with an Akt shRNA. **D** MDBK cells were pretreated with NF-κB inhibitor Bay 11-7085 and the cell lysates were collected individually at 4 hpi for immunoblotting with the respective antibodies. Signals were quantified using Image J software. The levels of the indicated proteins in the mock controls (0 h or un-treatment) were considered onefold. The protein levels were normalised to those for β-actin. The activation and inactivation folds indicated below each lane were normalized against values for the mock control. The predicted size of each protein was labeled at the right-hand side in kDa.

As shown above, BEFV-triggered autophagy benefits virus replication. Our previous study demonstrated that BEFV triggers the PI3K/Akt/NF-κB and Src/JNK/AP1 pathways in the stage of virus binding to cellular receptors to enhance cell entry of the virus [12]. In this study, we further examined activation of these pathways in the middle to late stages of the viral life cycle. MDBK cells were infected with BEFV at a MOI of 1 for different time periods and immunoblotted with the respective antibodies. PI3K was activated until 12 hpi and declined 18 hpi (Figure 4A). Akt displayed two peaks of activation (2 and 12 hpi) and declined 18 hpi (Figure 4B). Interestingly, decreased levels of the phosphorylated form of mTOR (p-mTOR) and ULK1 (p-ULK1) were seen at 18 hpi (Figure 4A). It is important to note that the accumulation of lipid conjugated LC3-II protein induced by BEFV became evident at 4 hpi and then slightly decreased at 18 hpi (Figure 4A). Furthermore, as compared with the mock-treated cells, we found that BEFV downregulated IκBα (Figures 4A and C). Since decreased levels of NF-κB subunit p50 and p65 were observed in the nucleus with the same kinetics pattern of Akt (Figure 4A), we next utilized an Akt shRNA to specifically target Akt. Our results revealed that the decreased levels of IκBα and NF-κB subunit p50 and p65 in the cytoplasm induced by BEFV were restored (Figure 4C), suggesting that Akt is the upstream inducer of NF-κB. Importantly, the BEFV-induced increase in the levels of LC3-II was also reversed in Akt knock-down cells (Figure 4C). To further confirm whether NF-κB is involved in BEFV-induced autophagy, an NF-κB inhibitor, Bay 11-7085, was used. Our results revealed that p-IkBα and LC3-II was partially reversed in BEFV-infected and Bay 11-7085-treated cells while the levels of Beclin 1, Atg 7, and p-ULK were not altered (Figure 4D). Our results further suggest that BEFV induces autophagy, at least in part, via the PI3K/Akt/NF-κB pathway.

**Figure 5:**
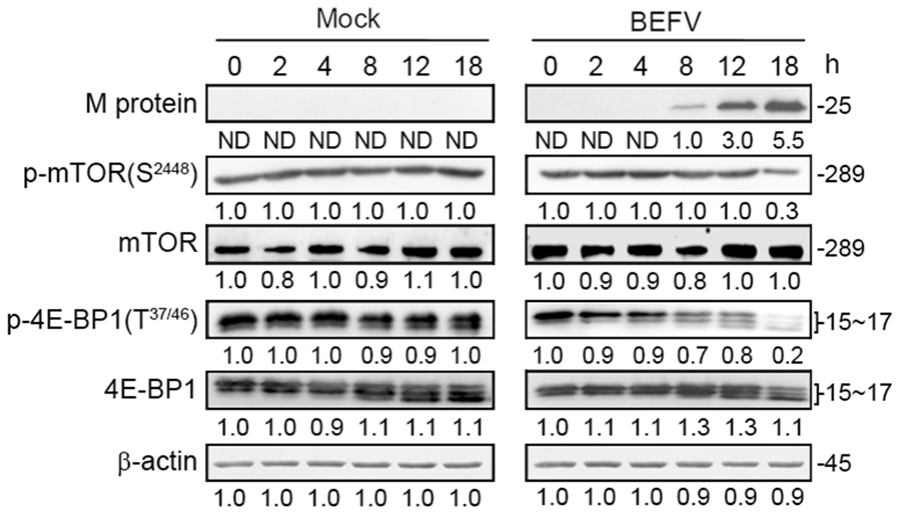
**BEFV reduces the phosphorylated form of mTOR in the late stage of infection.** MDBK cells were infected with BEFV at a MOI of 1 and the cell lysates were collected individually at the indicated time points for immunoblotting with the respective antibodies. Signals were quantified using Image J software. The levels of the indicated proteins at 0 h were considered onefold. The protein levels were normalised to those for β-actin. The activation and inactivation folds indicated below each lane were normalized against values for the 0 h. The predicted size of each protein was labeled at the right-hand side in kDa.

Having demonstrated that BEFV reduced the phosphorylated form of mTOR in the late stage of infection, we next wanted to confirm whether the downstream targets  were also suppressed. In the late stage of infection, the phosphorylated form of p-mTOR (S2448) as well as its downstream targets at different time points were analyzed. In cells with sufficient nutrients, mTOR is phosphorylated at Ser2448 via the PI3K/Akt pathway followed by the transmission of signals to inactivate the eIF4E inhibitor, 4E-BP1 [4]. Our results revealed that BEFV reduced the phosphorylated forms of mTOR and 4E-BP1 in the late stage of infection (Figures 4A and 5) as compared to mock-treated cells. Taken together, our results revealed that BEFV induced autophagy via inhibition of mTORC1 during the late stage of infection.

### BEFV triggers autophagy via activation of the Src/JNK/AP1 pathway

**Figure 6:**
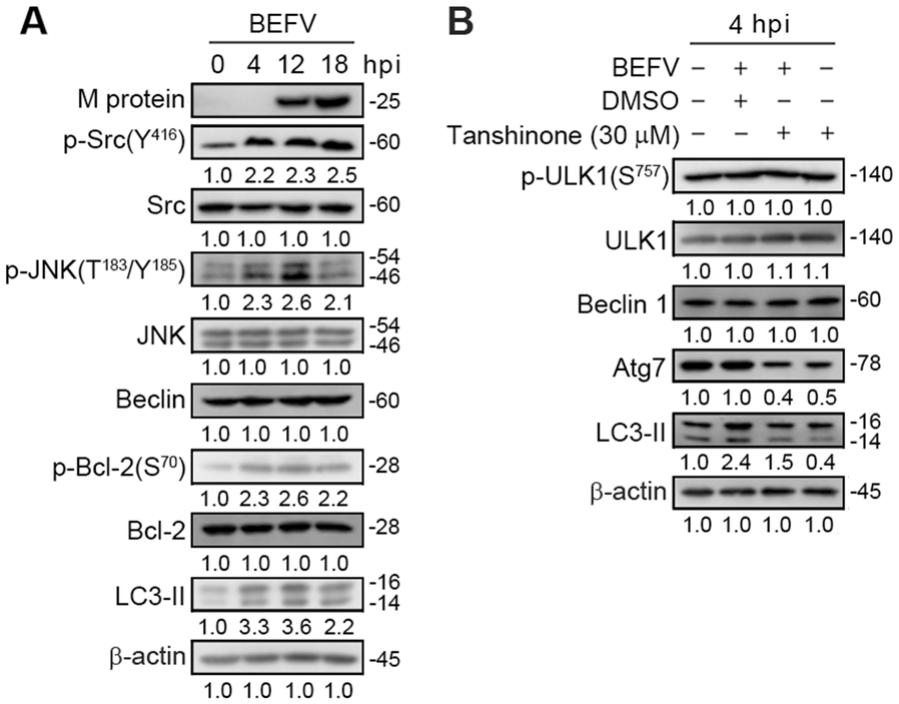
**BEFV induces autophagy through upregulation of the Src/JNK/AP-1 pathway. A** MDBK cells were infected with BEFV at a MOI of 1 and cell lysates were collected individually at the indicated time points for immunoblotting with the respective antibodies. **B** MDBK cells were pretreated with AP-1 inhibitor Tanshinone and the cell lysates were collected individually at 4 hpi for immunoblotting with the respective antibodies. The levels of the indicated proteins in the mock controls (0 h or un-treatment) were considered onefold. The protein levels were normalised to those for β-actin. The activation and inactivation folds indicated below each lane were normalized against values for the mock control.

Having demonstrated that BEFV-induced autophagy, at least in part, occurs via the PI3K/Akt/NF-κB pathway in the early to middle stages of infection. As shown in Figures 4A and 6A, an increase in the phosphorylated forms of Src and JNK occured in a time-dependent manner in MDBK cells in the early to late stages of BEFV infection. We next sought to determine whether the Src/JNK pathway is involved in BEFV-induced autophagy. It is important to note that an accumulation of lipid conjugated LC3-II protein induced by BEFV becomes evident at 4 hpi and then slightly decreased at 18 hpi (Figure 6A). The levels of Beclin 1 were not altered. Interestingly, the phosphorylated form of Bcl-2 and the level of LC3 II increased in a time-dependent manner in MDBK cells upon early to late stages of BEFV infection (Figure 6A). In Bay 11-7085-treated cells, the increased level of LC3-II was partially reversed in BEFV-infected cells (Figure 6B). The levels of Beclin 1 and p-ULK1 were not altered in Tanshinone-treated cells (Figure 6B) while the Atg 7 level was reduced (Figure 6B). Our results further suggest that BEFV-induced autophagy, at least in part, occurs via the Src/JNK/AP1 pathway. Collectively, the results suggest that BEFV triggers autophagy via simultaneous activation of the PI3K/Akt/NF-κB and Src/JNK/AP1 pathways.

### BEFV increases JNK1-mediated phosphorylation of Bcl-2 to release Beclin 1

**Figure 7:**
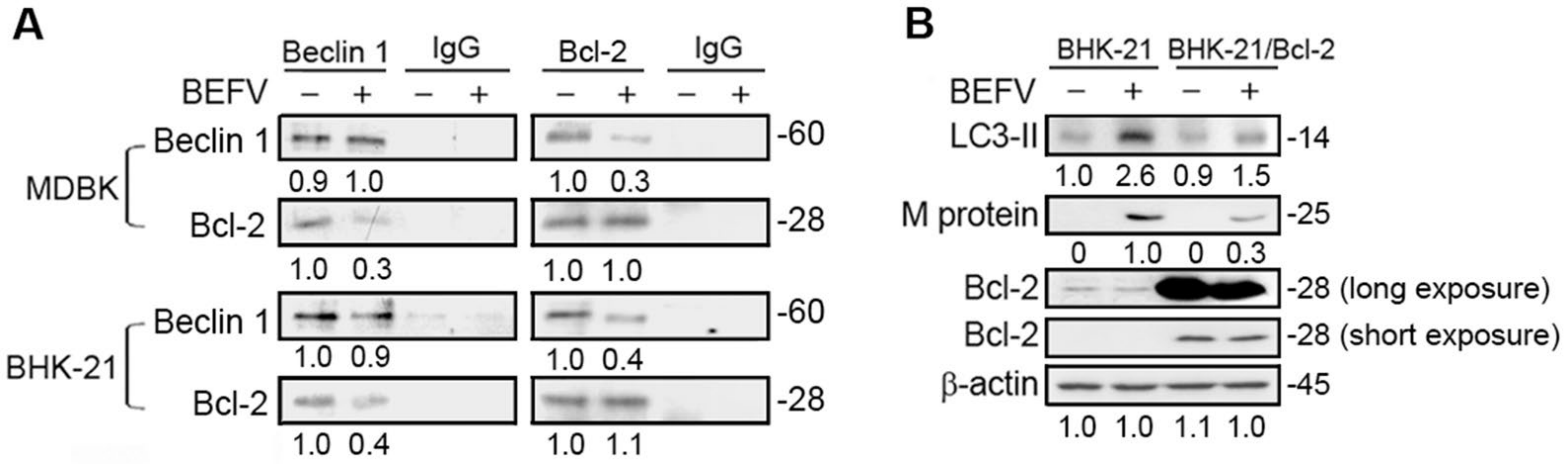
**BEFV inhibits Bcl-2 binding to Beclin 1. A** Cells were infected with BEFV at a MOI of 1 followed by reciprocal co-immunoprecipitation assays with the Bcl-2 or Beclin 1 antibodies. In reciprocal co-immunoprecipitation assays, anti-Beclin 1 rabbit IgG or anti-Bcl-2 mouse IgG were used as negative controls, respectively. **B** Overexpression of Bcl 2 suppresses BEFV production. BHK-21 and BHK-21/Bcl-2 cells were infected with BEFV at a MOI of 1. The cell lysate was collected at 18 hpi and subjected to immunoblotting for detecting the levels of M protein and LC3 II. The levels of the indicated proteins in the mock controls (un-infection) were considered onefold. The protein levels were normalised to those for β-actin. The activation and inactivation folds indicated below each lane were normalized against values for the mock control. Signals were quantified using Image J software. The predicted size of each protein was labeled at the right-hand side in kDa.

Pattingre and colleagues demonstrated that the antiapoptotic protein, Bcl-2, interacts with Beclin 1 and inhibits Beclin 1-dependent autophagy [32]. A recent study suggested that Akt-mediated regulation of autophagy and tumorigenesis through Beclin 1 phosphorylation [33]. An earlier study suggested that JNK1-mediated phosphorylation of Bcl-2 at Ser70, is required for starvation-induced autophagy [34]. It has been demonstrated that autophagy and apoptosis are basic cellular pathways that are regulated by JNK-mediated Bcl-2 phosphorylation [35]. JNK-mediated Bcl-2 phosphorylation inhibits its binding to Beclin1, which promotes the formation of autophagosomes [33]. Our results showed that BEFV activates the Src/JNK/AP1 pathway to increase JNK1-mediated phosphorylation of Bcl-2 at Ser70 (Figure 6A). Next, we analyzed the interaction between Beclin 1 and Bcl-2 during BEFV infection, since it is known that JNK activation triggered by environmental stimuli such as starvation can phosphorylate Bcl-2, leading to release of Bcl-2 from Beclin 1, thereby activating autophagy [34]. To understand whether BEFV can induce autophagy via regulating Bcl-2 activity, co-immunoprecipitation assays were carried out to evaluate the binding of Beclin 1 to Bcl-2. As shown in Figure 7A, co-immunoprecipitation assays confirmed that BEFV-modulated activation of JNK reduced the association of Beclin 1 and Bcl-2 in both MDBK and BHK-21 cells. To explore whether Bcl-2 plays a role in regulating BEFV-induced autophagy, a Bcl-2-overexpressing BHK-21 cell line was used [22]. As expected, overexpression of Bcl-2 suppressed BEFV-induced autophagy as evidenced by a reduction of LC3 II, leading to a decreased level of the BEFV M protein (Figure 7B). Our findings further confirm that BEFV increases JNK1-mediated phosphorylation of Bcl-2 to release Beclin 1, inducing autophagy.

### BEFV M protein induces autophagy by suppressing the PI3K/Akt/mTORC1 pathway in the late stage of infection

**Figure 8:**
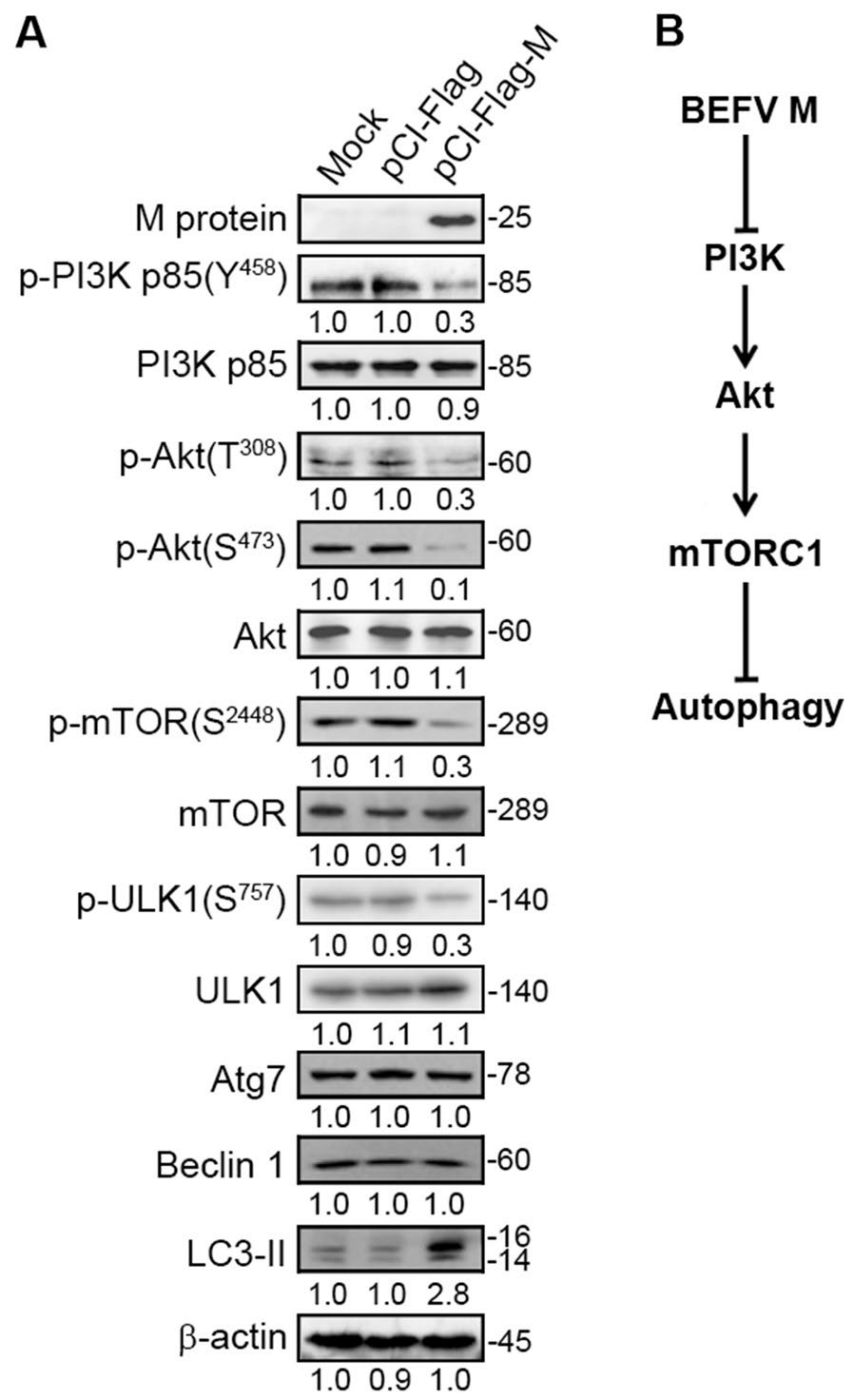
**The BEFV M protein induces autophagy through suppression of the PI3K/Akt/mTOR pathway. A** MDBK cells were transfected with pCI-Flag-M and the cell lysates were subjected to immunoblotting with the indicated antibodies. The levels of the indicated proteins in the mock control were considered onefold. The protein levels were normalised to those for β-actin. The activation and inactivation folds indicated below each lane were normalized against values for the mock control. Signals were quantified using Image J software. The predicted size of each protein was labeled at the right-hand side in kDa. **B** A model illustrating BEFV M protein-induced autophagy through suppression of the PI3K/Akt/mTOR pathway.

Transient expression of BEFV M protein alone in MDBK cells negatively regulated the PI3K/Akt/mTORC1 pathway, as revealed by analyzing the phosphorylated forms of PI3K (Y458), Akt (T308), and p-mTOR (S2448) as well as ULK1 (S757) which is a downstream phosphorylation target of mTOR to inhibit autophagy (Figure 8). On the other hand, the level of Akt phosphorylation in BEFV-infected MDBK cells showed a decrease after 18 hpi, while phosphorylation of Src and JNK increased (Figures 4A, B and 6A). Our data thus reveal that another viral protein may be involved in activating the PI3K/Akt/NF-κB pathway at the early stage of infection, while the BEFV M protein inhibits PI3K/Akt/mTORC1 from the middle to late stages, both of which induce autophagy.

## Discussion

Although activation of the PI3K/Akt/mTOR pathway in host cells is a crucial strategy for survival of a few viruses [10, 11, 36, 37], our results show that BEFV ingeniously inhibits mTORC1 but up-regulates the PI3K/Akt/NF-κB pathway to support viral multiplication. Our previous study revealed that BEFV raised Akt activity but conversely inactivated mTORC1 [10]. Although the inhibition of Akt reduced replication of BEFV, while inhibition of mTOR by rapamycin enhanced replication of the virus [10], the mechanisms underlying the PI3K/Akt/mTOR pathway-mediated BEFV replication remains unclear. More recently, we also showed that BEFV triggers simultaneously the PI3K/Akt/NF-κB and Src/JNK/AP1 pathways at the stage of virus binding to enhance virus entry [12]. This study further demonstrated that BEFV induces autophagy via upregulation of the PI3K/Akt/NF-κB and Src/JNK/AP1 pathways in the early to middle stages of infection and suppression of the PI3K/Akt/mTOR1 complex at the late stage of infection, thereby enhancing virus replication.

The PI3K/Akt pathway is an important mechanism through which viral infection influences various cell functions. Many viruses have been demonstrated to utilize the PI3K/Akt pathway to enhance their entry [12–16]. For example, the PI3K/Akt pathway controls vesicular trafficking of Ebola virus necessary for cell entry [16], while HSV triggers intracellular calcium release to promote viral entry by activating Akt signaling [13]. Our previous study demonstrated that BEFV triggers translocation of NF-κB subunits through the PI3K-Akt pathway to up-regulate NF-κB, enhancing cell entry [12], leading to productive infection. Unlike other virus such as VSV [12], the cell entry of BEFV depends on the PI3K-Akt signaling pathway.

Several viruses rely on activating the PI3K/Akt pathway for efficient multiplication or long-term persistence [10, 11, 36, 37]. Among various downstream factors, mTOR is an important mediator through which the PI3K/Akt pathway is linked to infection progress of some viruses [37, 38]. In the case of two persistently-infecting viruses, hepatitis B (HBV) and hepatitis C virus (HCV), it is known that they activate PI3K/Akt/mTOR signaling for cell survival and suppression of virus replication for long-term infection. Inhibition of mTOR slightly up-regulates replication of these two viruses [36, 37]. Non-segmented negativesense RNA viruses require Akt to enhance synthesis of viral RNAs [17]. Adenovirus relies on PI3K-mediated organization of actin filament for active internalization [38]. On the other hand, PI3K/Akt signaling is associated with up-regulation of the interferon response [38]. Higher PI3K-Akt activity might impede viral propagation through the activation of cellular defenses. Influenza A virus non-structural protein 1 (NS1) protein is an interesting case which activates the PI3K/Akt pathway to mediate antiapoptotic signaling responses [39]. Unlike most viruses, it was reported that the viral protein 1 (VP1) of foot-and-mouth disease virus inhibits Akt to promote cell death [38]. These reports confirm the multiple roles of the PI3K/Akt pathway in viral infection. We have previously demonstrated that BEFV-induced Akt phosphorylation is beneficial for BEFV replication [10]. Nevertheless, the role of the PI3K-Akt pathway in enhancing BEFV replication remained unknown until this work. This study demonstrates that BEFV activate NF-κB by promoting translocation of NF-κB subunits and by enhancing degradation of IκBα, which induces autophagy and enhances virus replication.

Central in the regulation of autophagy are two proteins, mTOR and Beclin 1 [40, 41]. In this study, depletion of Beclin 1 or overexpression of Bcl-2 reversed the BEFV-induced increase in LC3 II levels and leads to the significant reduction of virus yield. Immunoprecipitation assays revealed that BEFV disrupts the Beclin 1 and Bcl-2 interaction by JNK-mediated Bcl-2 phosphorylation, thereby activating autophagy. Our results suggest that BEFV stimulates the Src/JNK pathway to activate AP1 while suppressing Bcl 2, thereby inducing autophagy. mTOR is one of the key negative regulators of autophagy [41]. In the case of recently-discovered mTOR complexes, activation of mTORC1, which is directly up-regulated by the PI3K-Akt pathway, is required for such viruses. We demonstrated here for the first time that BEFV actively inhibits mTORC1 during the late stage of infection, thereby inducing autophagy. The compelling finding is that the ability of M protein to block the PI3K/Akt/mTORC1 pathway results in induction of autophagy. As mentioned above, HBV and HCV activate the PI3K/Akt pathway to slow down apoptosis and extend viral replication in both acute and persistent infection [36, 37]. Artificial inhibition of mTOR slightly up-regulates replication of these two viruses [36, 37]. Unlike HBV and HCV, BEFV possesses a different survival strategy as seen from up-regulation of Akt in the early to middle stage of infection but down-regulation of the function of the PI3K/Akt/mTORC1 pathway in the late stage of infection to support its multiplication. BEFV inhibits mTOR complex 1 to release 4E-BP and to induce autophage. Inhibition of the PI3K-Akt pathway decreases cap-dependent translation in several cell types including Vero cells [42]. Shutting off cell protein synthesis is another strategy that some viruses employ to escape host defenses or to increase the compatibility of their own transcripts [43, 44]. Inhibition of mTORC1 may therefore enhance BEFV multiplication through similar mechanisms. Notably, BEFV multiplication was not affected by down-regulation of the factors necessary for cap-dependent translation, as inhibition of mTORC 1 with rapamycin induced autophagy and increased viral yield. These results further suggest a critical role of autophagy in the virus life cycle, although we cannot rule out other effects on viral replication, independent of autophagy. In the case of VSV, it was demonstrated that the untranslated region of viral transcripts has a structure-dependent element for preferential translation [45], allowing us to speculate that BEFV could withstand inactivation of cap-dependent translation. In the present study, our results revealed that inhibition of autophagy by 3-MA or shRNA knock-down of cellular genes, including Beclin 1, ATG7, and LC3 which are important for the formation of autophagosomes, significantly reduced BEFV replication. This finding suggests that the autophagic machinery may play an important role in the replication of BEFV.

p62 is a multifunctional protein that associates with protein aggregates, interacts with LC3-II, and is selectively degraded by the autophagy-lysosome pathway [30]. p62 has also been considered a marker for autophagy-mediated protein degradation or autophagic flux. The expression levels of p62 and LC3-II were increased at 18 hpi and then decreased at 24 hpi as compared with mock infection, starvation or Thapsigarigin (TG) treatments. This suggests that BEFV may delay the degradation of p62 through an unknown mechanism before completion of virus replication. Moreover, a compelling observation is that knockdown of LAMP2, a critical protein responsible for the fusion of autophagosomes with lysosomes [28], significantly reduces virus replication. This suggests that autophagosome maturation likely plays a critical role in BEFV replication. Our recent report suggests that endosomal acidification is required for BEFV entry at a low pH [46]. The dependence of BEFV on the early and late endosomes indicates that virus is able to reach bona fide late endosomes for fusion and release from the endocytic pathway [46]. BEFV seems to have developed a strategy to promote formation and maturation of the autophagosome to support its own replication. The underlying mechanisms of the autophagosome benefiting virus replication will be addressed in the future. Conversely, several other viruses, such as coxsackieviruses, hepatitis C virus, influenza virus A, human immunodeficiency virus type 1 (HIV-1), and rotaviruses [47–50] have developed mechanisms to subvert autophagy by suppressing the fusion between autophagosomes and lysosomes.

**Figure 9:**
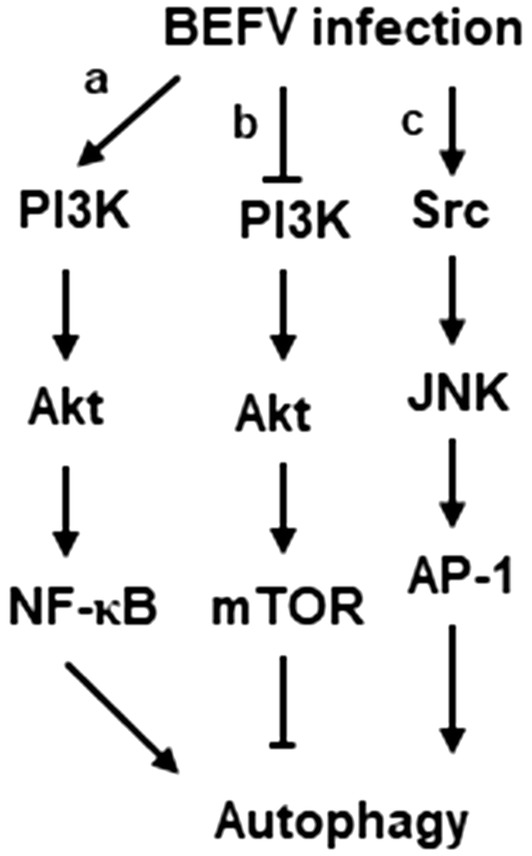
**A model illustrating BEFV-induced autophagy signaling pathways.** BEFV triggers autophagy to benefit viral production through either activation of the PI3K/Akt/NF-κB or Src/JNK/AP-1 pathways in the early to middle stages of infection (A, B) as well as suppression of the PI3K/Akt/mTOR complex 1 in the late stage of infection (C).

As shown in Figure 9, BEFV triggers autophagy to benefit viral replication through activation of multiple signaling pathways in the early to middle stages of infection and the late stage of infection. This study provides new insights into the underlying mechanisms of BEFV-induced autophagosome formation, broadens our understanding of BEFV-induced autophagy enhancing virus replication and may facilitate new strategies for combating BEFV-caused diseases.

## Supplementary information

**Additional file 1. Cell viability in drug-treated (A) or shRNA-knock down (B) cells.** To examine whether shRNAs and compounds used in this work have deleterious effects on the cell, cell viability was examine by MTT assay.

## References

[1] De Virgilio C, Loewith R (2006). The TOR signalling network from yeast to man. Int J Biochem Cell Biol.

[2] Bader AG, Kang S, Zhao L, Vogt PK (2005). Oncogenic PI3K deregulates transcription and translation. Nat Rev Cancer.

[3] Gingras A, Kennedy SG, O’Leary MA, Sonenberg N, Hay N (1998). 4E-BP1, a repressor of mRNA translation, is phosphorylated and inactivated by the Akt (PKB) signaling pathway. Genes Dev.

[4] Hirsch E, Costa C, Ciraolo E (2007). Phosphoinositide 3-kinases as a common platform for multi-hormone signaling. J Endocrinol.

[5] Alessi DR, Andjelkovic M, Caudwell B, Cron P, Morrice N, Cohen P, Hemmings BA (1996). Mechanism of activation of protein kinase B by insulin and IGF-1. EMBO J.

[6] Sarbassov DD, Guertin DA, Ali SM, Sabatini DM (2005). Phosphorylation and regulation of Akt/PKB by the rictor–mTOR complex. Science.

[7] Liu P, Begley M, Michowski W, Inuzuka H, Ginzberg M, Gao D, Tsou P, Gan W, Papa A, Kim BM, Wan L, Singh A, Zhai B, Yuan M, Wang Z, Gygi SP, Lee TH, Lu KP, Toker A, Pandolfi PP, Asara JM, Kirschner MW, Sicinski P, Cantley L, Wei W (2014). Cell-cycle-regulated activation of Akt kinase by phosphorylation at its carboxyl terminus. Nature.

[8] Huang WR, Chi PI, Chiu HC, Hsu JL, Nielsen BL, Liao TL, Liu HJ (2017). Avian reovirus p17 and σA act cooperatively to downregulate Akt by suppressing mTORC2 and CDK2/cyclinA2 and upregulating proteasome subunit PSMB6. Sci Rep.

[9] Kimura N, Tokunaga C, Dalal S, Richardson C, Yoshino K, Hara K, Kemp BE, Witters LA, Mimura O, Yonezawa K (2003). A possible linkage between AMP-activated protein kinase (AMPK) and mammalian target of rapamycin (mTOR) signalling pathway. Genes Cells.

[10] Ji WT, Wang YC, Lin FL, Liao MH, Shih WL, Liu HJ (2011). Inhibitors of phosphatidylinositol 3-kinase and mTOR but not Akt enhance replication of bovine ephemeral fever virus. Vet J.

[11] Cooray S (2004). The pivotal role of phosphatidylinositol 3-kinase-Akt signal transduction in virus survival. J Gen Virol.

[12] Cheng CY, Huang WR, Chi PI, Chiu HH, Liu HJ (2015). Cell entry of bovine ephemeral fever virus requires activation of Src-JNK-AP1 and PI3K-Akt-NFκB pathways as well as Cox-2-mediated PGE2/EP receptor signaling to enhance clathrin-mediated virus endocytosis. Cell Microbiol.

[13] Cheshenko N, Trepanier JB, Stefanidou M, Buckley N, Gonzalez P, Jacobs W, Herold BC (2013). HSV activates Akt to trigger calcium release and promote viral entry: novel candidate target for treatment and suppression. FASEB J.

[14] Feng SZ, Cao WS, Liao M (2011). The PI3K/Akt pathway is involved in early infection of some exogenousavian leukosis viruses. J Gen Virol.

[15] Liu Z, Tian Y, Machida K, Lai MM, Luo G, Foung SK, Ou JH (2012). Transient activation of the PI3K-AKT pathway by hepatitis C virus to enhance viral entry. J Biol Chem.

[16] Saeed MF, Kolokoltsov AA, Freiberg AN, Holbrook MR, Davey RA (2008). Phosphoinositide-3 kinase-Akt pathway controls cellular entry of Ebola virus. PLoS Pathog.

[17] Sun M, Fuentes SM, Timani K, Sun D, Murphy C, Lin Y, August A, Teng MN, He B (2008). J Virol.

[18] Holmes IH, Doherty RL (1970). Morphology and development of bovine ephemeral fever virus. J Virol.

[19] Walker PJ, Byrne KA, Cybinski DH, Doolan DL, Wang Y (1991). Proteins of bovine ephemeral fever virus. J Gen Virol.

[20] Harty RN, Paragas J, Sudol M, Palese P (1999). A proline-rich motif within the matrix protein of vesicular stomatitis virus and rabies virus interacts with WW domains of cellular proteins: implications for viral budding. J Virol.

[21] Harty RN, Brown ME, McGettigan JP, Wang G, Jayakar HR, Huibregtse JM, Whitt MA, Schnell MJ (2001). Rhabdoviruses and the cellular ubiquitin-proteasome system: a budding interaction. J Virol.

[22] Chulu JL, Lee LH, Lee YC, Liao SH, Lin FL, Shih WL, Liu HJ (2007). Apoptosis induction by avian reovirus through p53 and mitochondria-mediated pathway. Biochem Biophy Res Comm.

[23] Schneider CA, Rasband WS, Eliceiri KW (2012). NIH Image to ImageJ: 25 years of image analysis. Nat Methods.

[24] Tanida I, Ueno T, Kominami E (2004). LC3 conjugation system in mammalian autophagy. Int J Biochem Cell Biol.

[25] Rubinsztein DC, Gestwicki JE, Murphy LO, Klionsky DJ (2007). Potential therapeutic applications of autophagy. Nat Rev Drug Discov.

[26] Seglen PO, Gordon PB (1982). 3-Methyladenine. Specific inhibitor of autophagic/lysosomal protein degradation in isolated rat hepatocytes. Proc Natl Acad Sci USA.

[27] Klionsky DJ, Emr SD (2000). Autophagy as a regulated pathway of cellular degradation. Science.

[28] Klionsky DJ (2007). Autophagy. From phenomenology to molecular understanding in less than a decade. Nat Rev Mol Cell Biol.

[29] Saftig P, Beertsen W, Eskelinen EL (2008). LAMP-2.Acontrol step for phagosome and autophagosome maturation. Autophagy.

[30] Seibenhener ML, Babu JR, Geetha T, Wong HC, Krishna NR, Wooten MW (2004). Sequestosome 1/p62 is a polyubiquitin chain-binding protein involved in ubiquitin proteasome degradation. Mol Cell Biol.

[31] Kimura S, Noda T, Yoshimori T (2007). Dissection of the autophagosome maturation process by a novel reporter protein, tandem fluorescent-tagged LC3. Autophagy.

[32] Pattingre S, Tassa A, Qu S, Garuti R, Liang XH, Mizushima N, Packer M, Schneider MD, Levine B (2005). Bcl-2 antiapoptotic proteins inhibit Beclin 1-dependent autophagy. Cell.

[33] Wang RC, Wei Y, An Z, Zou Z, Xiao G, Bhagat G, White M, Reichelt J, Levine B (2012). Akt-mediated regulation of autophagy and tumorigenesis through Beclin 1 phosphorylation. Science.

[34] Wei Y, Sinha S, Levine B (2008). Dual role of JNK1-mediated phosphorylation of Bcl-2 in autophagy and apoptosis regulation. Autophagy.

[35] Wirawan E, Walle LV, Kersse K, Cornelis S, Claerhout S, Vanoverberghe I, Roelandt R, De Rycke R, Verspurten J, Declercq W, Agostinis P, Vanden Berghe T, Lippens S, Vandenabeele P (2010). Caspase-mediated cleavage of Beclin-1 inactivates Beclin-1-induced autophagy and enhances apoptosis by promoting the release of proapoptotic factors from mitochondria. Cell Death Dis.

[36] Guo H, Zhou T, Jiang D, Guconati A, Xiao GH, Block TM, Guo JT (2007). Regulation of hepatitis B virus replication by the phosphatidylinositol 3-kinase-akt signal transduction pathway. J Virol.

[37] Mannova P, Beretta L (2005). Activation of the N-Ras-PI3K-Akt-mTOR pathway by hepatitis C virus: control of cell survival and viral replication. J Virol.

[38] Ji WT, Liu HJ (2008). PI3K-Akt signaling and viral infection. Recent Pat Biotechnol.

[39] Ehrhardt C, Wolff T, Pleschka S, Planz O, Beermann W, Bode JG, Schmolke M, Ludwig S (2007). Influenza A virus NS1 protein activates the PI3K/Akt pathway to mediate antiapoptotic signaling responses. J Virol.

[40] Kihara A, Kabeya Y, Ohsumi Y, Yoshimori T (2001). Beclin-phosphatidylinositol 3-kinase complex functions at the trans-Golgi network. EMBO Rep.

[41] Codogno P, Meijer AJ (2005). Autophagy and signaling. Their role in cell survival and cell death. Cell Death Differ.

[42] Edgil D, Polacek C, Harris E (2006). Dengue virus utilizes a novel strategy for translation initiation when cap-dependent translation is inhibited. J Virol.

[43] Lyles DS (2000). Cytopathogenesis and inhibition of host gene expression by RNA viruses. Microbiol Mol Biol Rev.

[44] Lopez-Lastra M, Rivas A, Barria MI (2005). Protein synthesis in eukaryotes: the growing biological relevance of cap-independent translation initiation. Biol Res.

[45] Whitlow ZW, Connor JH, Lyles DS (2006). Preferential translation of vesicular stomatitis virus mRNAs is conferred by transcription from the viral genome. J Virol.

[46] Cheng CY, Shih WL, Huang WR, Chi PI, Wu MH, Liu HJ (2012). Bovine ephemeral fever virus uses a clathrin-mediated and dynamin 2-dependent endocytic pathway that requires Rab5 and Rab7 as well as microtubules for endocytosis. J Virol.

[47] Jackson WT, Giddings TH, Taylor MP, Mulinyawe S, Rabinovitch M, Kopito RR, Kirkegaard K (2005). Subversion of cellular autophagosomal machinery by RNA viruses. PLoS Biol.

[48] Wong J, Zhang J, Si X, Gao G, Mao I, McManus BM, Luo H (2008). Autophagosome supports coxsackievirus B3 replication in host cells. J Virol.

[49] Sir D, Chen WL, Choi J, Wakita T, Yen TS, Ou JH (2008). Induction of incomplete autophagic response by hepatitis C virus via the unfolded protein response. Hepatology.

[50] Gannagé M, Dormann D, Albrecht R, Dengjel J, Torossi T, Rämer PC, Lee M, Strowig T, Arrey F, Conenello G, Pypaert M, Andersen J, García-Sastre A, Münz C (2009). Matrix protein 2 of influenza A virus blocks autophagosome fusion with lysosomes. Cell Host Microbe.

