# Supplementary figures and images for: Bovine ephemeral fever virus triggers autophagy enhancing virus replication via upregulation of the Src/JNK/AP1 and PI3K/Akt/NF-κB pathways and suppression of the PI3K/Akt/mTOR pathway
Source: Vet Res. 2019 Oct 10;50:79. doi: 10.1186/s13567-019-0697-0 (PMC6785866; doi:10.1186/s13567-019-0697-0)

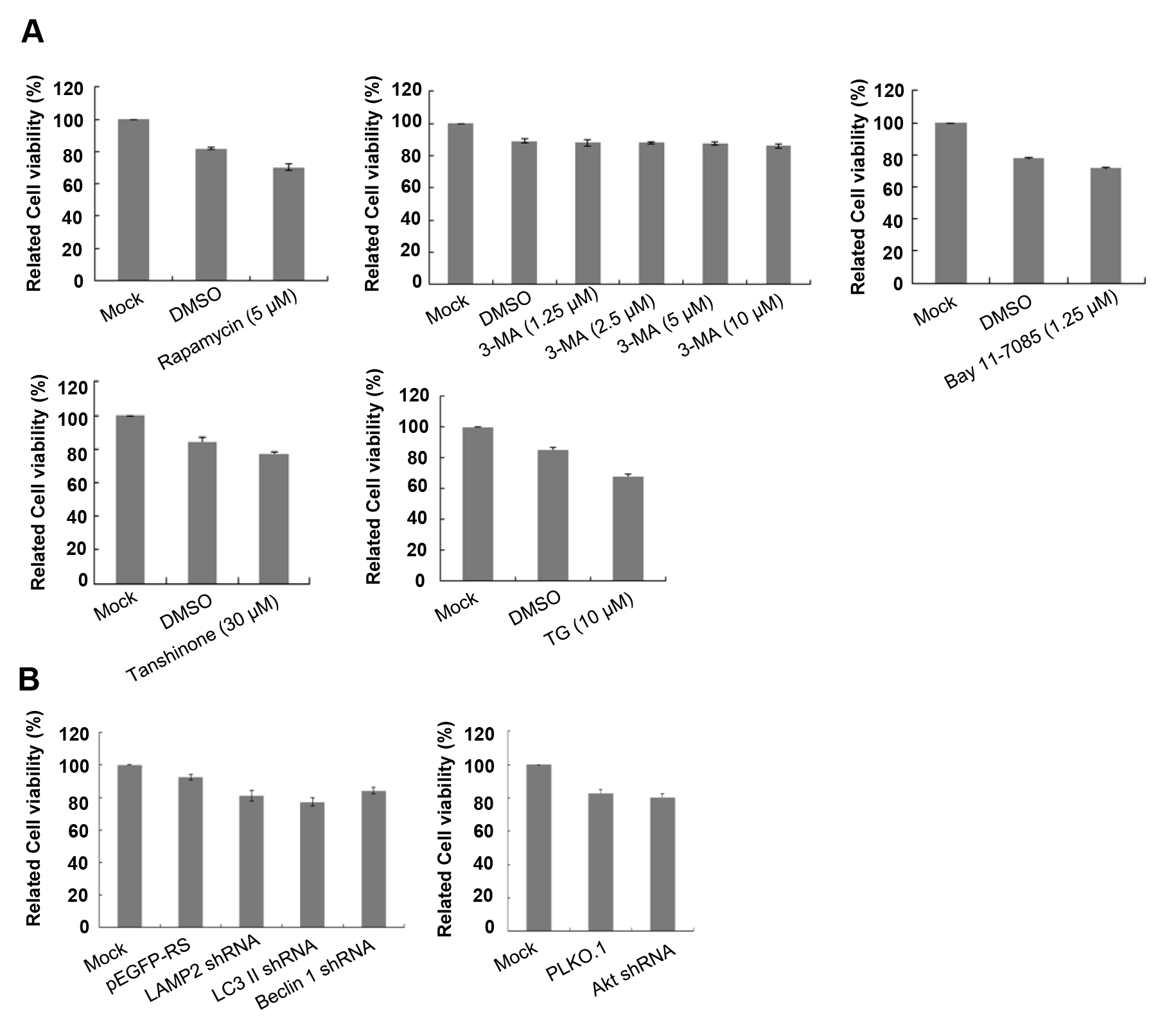

Supplement: Supplementary file 1 — Additional file 1. Cell viability in drug-treated (A) or shRNA-knock down (B) cells. To examine whether shRNAs and compounds used in this work have deleterious effects on the cell, cell viability was examine by MTT assay. [file 13567_2019_697_MOESM1_ESM.tif]
